# Interactive, Visual Simulation of a Spatio-Temporal Model of Gas Exchange in the Human Alveolus

**DOI:** 10.3389/fbinf.2021.774300

**Published:** 2022-01-26

**Authors:** Kerstin Schmid, Andreas Knote, Alexander Mück, Keram Pfeiffer, Sebastian von Mammen, Sabine C. Fischer

**Affiliations:** ^1^ Supramolecular and Cellular Simulations, Center for Computational and Theoretical Biology, Faculty of Biology, University of Würzburg, Würzburg, Germany; ^2^ Human Computer Interaction, Institute of Computer Science, Faculty of Mathematics and Computer Science, University of Würzburg, Würzburg, Germany; ^3^ Behavioral Physiology and Sociobiology, Biocenter, Faculty of Biology, University of Würzburg, Würzburg, Germany

**Keywords:** interactive simulation, visualization, theoretical modeling, lung physiology, requirements analysis, spatio-temporal resolution, education

## Abstract

In interdisciplinary fields such as systems biology, good communication between experimentalists and theorists is crucial for the success of a project. Theoretical modeling in physiology usually describes complex systems with many interdependencies. On one hand, these models have to be grounded on experimental data. On the other hand, experimenters must be able to understand the interdependent complexities of the theoretical model in order to interpret the model’s results in the physiological context. We promote interactive, visual simulations as an engaging way to present theoretical models in physiology and to make complex processes tangible. Based on a requirements analysis, we developed a new model for gas exchange in the human alveolus in combination with an interactive simulation software named *Alvin*. *Alvin* exceeds the current standard with its spatio-temporal resolution and a combination of visual and quantitative feedback. In *Alvin*, the course of the simulation can be traced in a three-dimensional rendering of an alveolus and dynamic plots. The user can interact by configuring essential model parameters. *Alvin* allows to run and compare multiple simulation instances simultaneously. We exemplified the use of *Alvin* for research by identifying unknown dependencies in published experimental data. Employing a detailed questionnaire, we showed the benefits of *Alvin* for education. We postulate that interactive, visual simulation of theoretical models, as we have implemented with *Alvin* on respiratory processes in the alveolus, can be of great help for communication between specialists and thereby advancing research.

## 1 Introduction

Systems biology is a highly interdisciplinary research field that integrates theoretical modeling and experimental data (Gavaghan et al., 2006). A key component of projects with valuable scientific progress is close cooperation between experimentalists and theorists (Byrne et al., 2006; Drubin and Oster, 2010; Welsh et al., 2006). However, this entails certain challenges. Different ways of thinking and terminologies or jargon often hinder communication between the disciplines. Ongoing efforts to bridge the gap include educational reviews [e.g., (Sharpe, 2017; Fischer, 2019)], summer schools, special research programs (https://www.newton.ac.uk/event/cgp/) and large multi-laboratory initiatives such as the Virtual Physiological Human (Viceconti et al., 2008) or The Virtual Brain (https://www.thevirtualbrain.org). Key components of these approaches are informative visualizations and the possibility of hands-on experience.

The goal of our study was to create a tool to better present modeling results to experimenters. To this end, we consider communicating results of mathematical modeling in physiology. In publications, models are usually presented as follows (Mogilner et al., 2011): The model definition is given in terms of mathematical equations, occasionally supported by schematic diagrams describing the model structure. For the corresponding simulations, all parameter values are listed and the output is visualized in graphs and compared with experimental data, where appropriate. When modeling spatial structures and processes, the simulation output is presented in still images or, if possible, animations (Chao, 2003; Lin et al., 2004; Saber and Heydari, 2012). As an alternative for the communication of state of the art theoretical models, we promote interactive, visual simulation. Previous approaches include computer-aided diagnosis software (Xiong et al., 2017; Conover et al., 2018) or systems for medical education (Jacob et al., 2012; Jamniczky et al., 2012; Costabile, 2021). We focus on the human lung. Existing interactive systems for teaching in this field address respiratory mechanics (Kuebler et al., 2007; Warliah et al., 2012) or gas exchange (Kapitan, 2008). All above systems for teaching convey established educational content. They have not been intended to advance the current state of research. In contrast, (Winkler et al., 1995) argue that their interactive system has great utility beyond its educational use. They have developed an application that provides an interactive interface with a simulation of a multi-compartment model. Ventilation mechanics, gas transport, gas mixing and gas exchange are considered. However, the actual process of gas exchange, the key functionality of the human lung, remains as abstract as the site where it occurs.

We thus focused on the smallest functional unit of the lung - the alveolus. The overarching goal was to provide an interactive visualization of the process of gas exchange in the human alveolus for research and education. We refined and combined existing models (Weibel et al., 1993; Dash et al., 2016) to cover the complete transport of oxygen into hemoglobin. The resulting model provided the computational core for an interactive simulation software named *Alvin*. *Alvin* facilitates investigations of relationships between morphological and physiological factors and the course of gas exchange. The software enables systematic investigations of our model with respect to experimental data. We aimed to maximize the usability of *Alvin* for both research-related and educational usage. As an exemplary use case in research, we present a plausibility check of pulmonary diffusion capacity measurements. Concerning the applicability of *Alvin* in teaching, we present the details of its integration into a digital physiology lab course for undergraduate students and the results of a corresponding survey among its participants. The software is available for download at https://go.uniwue.de/alvin.

Particular about our work is the development of the mathematical model with the aim of visualization in combination with the requirements-based engineering of the simulation software. This resulted in an advanced gas exchange model and an interactive application that exceed the existing standard. Specifically, design features as the ability to run and compare multiple simulation instances at the same time and the combination of providing parameter value presets as well as allowing parameter configurations by the user are key contributions to the field. This results in an educationally valuable application that also allows revealing unknown underlying assumptions of results presented in the literature. Taken together, our work demonstrates that an interactive, visual simulation is a versatile and powerful tool to visualize modeling results for both researchers and students.

## 2 Methods

On the basis of our goals, corresponding requirements were defined in a user-centered engineering approach. Our interdisciplinary team included a development team (AK, AM, KS) and supervising experts (SvM for games engineering, SCF for mathematical modelling, KP for physiology education). Concepts on requirements were first drafted within the development team. These concepts were then either acknowledged by experts/stakeholders in a quality gateway or returned for revision. The higher-level requirements could be categorized into three groups: Scientific (S), educational (E) and accessibility (A) requirements.

S.1. Gas exchange model suitable for interactive configuration.
S.2. Interfaces for interaction.
S.3. Quantitative simulation output.
S.4. Visual feedback that emphasizes the connection between structure and function of the alveolus.
E.1. Presentation of educationally relevant respiratory phenomena.
E.2. Facilitate autonomous work with the application.
A.1. Compatibility with common devices (computers or tablets with windows, iOS or linux).
A.2. Simple and clear GUI (to enhance the intuitive use of the system).
A.3. Applicability to the widest possible range of scientific issues.

In an iterative process, system requirements and final design requirements were developed from these higher-level user requirements (and recorded in a total of 166 GitLab issues). The complete set of requirements is listed in Section S1.1 of the Supplementary Material.

## 3 Results and Discussion

### 3.1 Integrative Alveolar Gas Exchange Model

The human lung consists of progressively branching bronchi and bronchioles, and blood vessels follow this structure (Hsia et al., 2016). The respiratory zone begins where the first alveoli adjoin the bronchioles (Haefeli-Bleuer and Weibel, 1988). Alveoli are hollow protrusions that have a large surface area and a thin tissue barrier. They are surrounded by a dense network of fine capillaries (Weibel and Gomez, 1962). Within an alveolus, inhaled air passes through the cavity and gas exchange with the capillary blood takes place through the tissue barrier (Weibel, 2009). An alveolus thus represents the smallest functional unit of the lung. We established a spatio-temporal model of gas exchange in the human alveolus based on empirically established models (Weibel et al., 1993; Dash et al., 2016) (requirement S.1). This entailed the integration of the established models and the alignment of their numerical scales. Any gaps in the model had to be identified and closed. Finally, the new model was validated against data from the literature.

#### 3.1.1 Model

The process of gas exchange in an alveolus can be divided into two sequential steps (Roughton and Forster, 1957): 1. The diffusion of oxygen through the tissue barrier into the blood and red blood cells and 2. its binding to hemoglobin (Hb). For each step, we adopted an established model describing this process (Weibel et al., 1993; Dash et al., 2016). By integrating the two sub-models into a complete model we can simulate the entire process of gas exchange inside an alveolus. The diffusion of oxygen across the alveolar wall is calculated based on Fick’s law (Weibel et al., 1993), resulting in

$$\nu ={DMO}_{2}\cdot \Delta pO_{2}=K_{O_{2}}\cdot \frac{s}{\tau }\cdot {\Delta pO}_{2}$$ (1)

**FIGURE 1:**
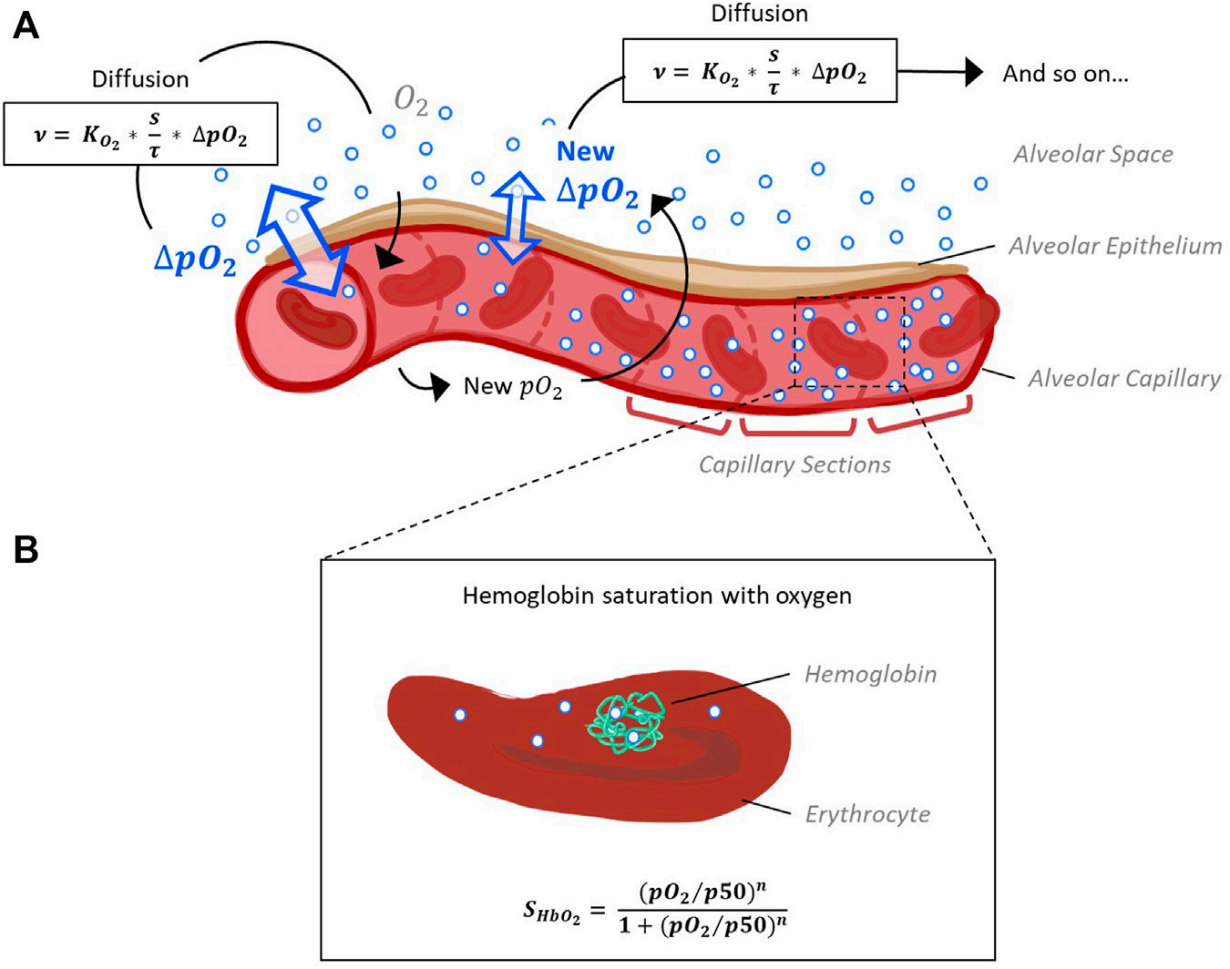
Schematic representation of the model capillary with erythrocytes, separated from alveolar space by a single cell layer of alveolar epithelium. **(A)** In order to reconstruct O_2_ and CO_2_ pressure gradients along the capillary, it is divided into sections of equal size. The pressure gradient between alveolar space and blood (ΔpO_2_) and the resulting flow of oxygen along this gradient is calculated for each section subsequently, as oxygen flow into one section affects pO_2_ and thus ΔpO_2_ of the next section. Calculation of oxygen diffusion depending on ΔpO_2_ is based on Fick’s law (Weibel et al., 1993). **(B)** According to the pO_2_ and pCO_2_ gradients along the capillary sections determined in step 1, hemoglobin oxygen saturation (S_HbO2_) is calculated for each section. The corresponding Hill equation has been defined and fitted to experimental data (Dash et al., 2016).

The oxygen flow *ν* across the barrier is a function of the pressure gradient ΔpO_2_ between air and blood and morphological parameters that contribute to the so called membrane diffusing capacity for oxygen DMO_2_. More precisely, DMO_2_ comprises the ratio between surface area *s* and barrier thickness *τ* multiplied by the permeability coefficient $K_{O_{2}}$ . Standing alone, this calculation would yield a mean quantity of oxygen flow in the alveolus. However, the potential of visualization should be exploited and the course of diffusion along the capillary should be shown in the alveolar model. This is particularly interesting as partial pressures of respiratory gases inside the blood are not homogeneous in the alveolar region. Gas exchange leads to oxygen (O_2_) and carbon dioxide (CO_2_) pressure gradients in the alveolar capillary. In a healthy individual, blood enters this area with a low partial pressure of oxygen (pO_2_) and a high partial pressure of carbon dioxide (pCO_2_). Diffusion of O_2_ from the alveolus into the capillary and of CO_2_ out of the capillary into the alveolus gradually increases pO_2_ and decreases pCO_2_ until the distribution of gases reaches equilibrium (Powers and Dhamoon, 2019). Hence, the course of pressure gradients depends on the efficiency of gas diffusion and the blood flow velocity. To map O_2_ and CO_2_ pressure gradients in our model, a representative capillary was divided into subsections of equal size (Figure 1). Oxygen diffusion from the alveolar space into the different sections is calculated successively starting with the first section. Here, blood enters with a preset pO_2_. This involves a partial pressure gradient with respect to the alveolar space. The diffusion along this gradient is calculated according to Eq. 1. The absolute amount of oxygen that reaches this capillary section is calculated from this oxygen flow and the blood flow velocity. It affects the pO_2_ of the blood in the next section, which is considered in a new calculation cycle and so on.

The quantity of CO_2_ diffusing out of the capillary and into the alveolus is determined via the respiratory exchange ratio from the quantity of oxygen that is taken up by the blood. The respiratory exchange ratio is defined as the amount of CO_2_ produced divided by the amount of O_2_ consumed. This ratio is assessed by analyzing exhaled air in comparison with the environmental air and its average value for the human diet is around 0.82 (Sharma et al., 2020). Taken together, this provides a time-resolved model for the first step of gas exchange: The diffusion of oxygen from inhaled air into the capillary blood of the alveolus and of carbon dioxide in the reverse direction.

In a second step, the binding of O_2_ and CO_2_ to hemoglobin was adopted from (Dash et al., 2016), such that

$$S_{{\text{HbO}}_{2}}=\frac{{\left({pO}_{2}/p50\right)}^{nH}}{1+{\left({pO}_{2}/p50\right)}^{nH}}$$ (2)

Hemoglobin oxygen saturation (S_HbO2_) is expressed as a Hill function depending on pO_2_, the Hill coefficient *nH* and *p*50, the value of pO_2_ at which hemoglobin is 50% saturated with O_2_. The parameter *nH*, in turn, depends on pO_2_. Polynomial expressions describe the dependence of *p*50 on pCO_2_ in the blood, blood temperature, the pH inside erythrocytes (pH_rbc_) and concentration of the organic phosphate 2,3-bisphosphoglycerate ([2,3]-DPG). These dependencies have been described and fitted to several experimental data sets (Dash et al., 2016) for a wide range of parameter values (fulfills requirement A.3.1). In our model, S_HbO2_ is calculated for each section according to the pO_2_ and pCO_2_ gradients along the capillary sections determined in step 1. Hence, we obtain the distribution of blood oxygen saturation along the capillary as the main output of our model.

Together, this yields a model for the complete process of oxygen transport from inhaled air into hemoglobin in the blood with spatio-temporal resolution. All parameters essential for the model and their default values were collected from the literature and represent a normal, healthy condition (Table 1).

**TABLE 1:** Model parameters and their default values. Values of morphological and physiological parameters of the gas exchange model were collected from literature. All values given are mean values referring to a single alveolus.

Parameter	Unit	Default value	References	Value range
Alveolar pO_2_	mmHg	100	Sharma et al. (2020)	1–150
Blood pO_2_	mmHg	40	Dash et al. (2016)	1–150
Alveolar pCO_2_	mmHg	40	Sharma et al. (2020)	1–150
Blood pCO_2_	mmHg	45	Dash et al. (2016)	1–150
Surface area	μm^2^	121,000	Mercer et al. (1994)	0–210 000
Thickness of tissue barrier	μm	1.11	Gehr et al. (1978); Weibel et al. (1993)	0.1–3.0
Blood flow velocity	mm/s	1	Abstracted from: Weibel et al. (1993); Petersson and Glenny, (2014)	0.01–2
Blood volume	μm^3^	404,000 (50% “capillary recruitment”)	Abstracted from: Gehr et al. (1978); Ochs et al. (2004); Okada et al. (1992)	1–808,000
Blood temperature	°C	37	Dash et al. (2016)	20–44
Erythrocyte pH (pH_rbc_)		7.24	Dash et al. (2016)	5.8–8.2
Concentration of [2,3]-DPG	mM	4.65	Dash et al. (2016)	1–10
Capillary length	μm	500	Weibel et al. (1993)	*not adjustable
Capillary volume	μm^3^	808,000	Ochs et al. (2004); Gehr et al. (1978)	*not adjustable
Capillary radius	μm	3.15	Mühlfeld et al. (2010)	*not adjustable
Number of capillaries		52	Calculated from capillary volume, radius and length	*not adjustable

#### 3.1.2 Model Validation

**FIGURE 2:**
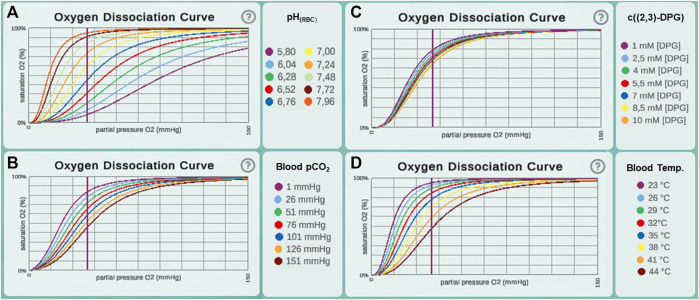
Oxygen dissociation curves recreated in *Alvin* for different ranges of parameter values from the original paper (Dash et al., 2016). This includes value ranges for the parameters **(A)** pH in erythrocytes (pH_rbc_), **(B)** blood pCO_2_, **(C)** concentration of [2,3]-DPG and **(D)** blood temperature.

In a first step of model validation, we analysed whether the two sub models from step 1 and step 2 had been sensibly adapted from the literature. In our model, oxygen diffusion is estimated for a single alveolus with a surface area of 121,000 μm^2^. Other parameters affecting DMO_2_ (namely tissue barrier thickness and permeability coefficient, see Eq. 1) were adopted without change. DMO_2_ of the whole lung in relation to body weight (bw) was estimated as 0.079 ml/(s × mmHg × kg) (Weibel et al., 1993). To compare our model result (DMO_2_^(model)^ = 6 × 10^–9^ ml/(s × mmHg)) with Weibel’s estimate, it needs to be extrapolated to the organ scale. Multiplying DMO_2_^(model)^ by the number of alveoli in the human lung (480 × 10^6^ (Ochs et al., 2004)) results in a DMO_2_^(model, extrapolated)^ of 2.88 ml/(s × mmHg). This value is distinctly lower than the DMO_2_ estimated by Weibel et al., assuming a standard body weight of 70 kg: DMO_2_^(Weibel, bw 70 kg)^ = 5.53 ml/(s × mmHg). This estimate has been based on morphometric studies in fully inflated, fluid-filled lungs (Weibel et al., 1993). It is recognized that in an air-filled lung, however, only about 60–70% of the alveolar surface is exposed to air (Gil et al., 1979; Bachofen et al., 1987). The default value for surface area in our model was taken from studies on perfusion-fixed, air-filled lungs (Mercer et al., 1994). Hence, our combination of parameter values for the surface area of a single alveolus (Mercer et al., 1994) and the number of alveoli in the human lung (Ochs et al., 2004) produce a result that falls short of the previous estimate. However, the discrepancy is explained by known differences in the morphometric methods used. We deliberately chose the surface value from the study on an air-filled lung to be as close as possible to the *in vivo* situation. The sub model describing hemoglobin oxygen saturation was adopted from the literature (Dash et al., 2016) without further modifications. Hb-O_2_ dissociation curves across the different parameter ranges from this publication [Figure 4 E-H in (Dash et al., 2016)] were recreated and indicate a correct implementation of the model (Figure 2).

**FIGURE 3:**
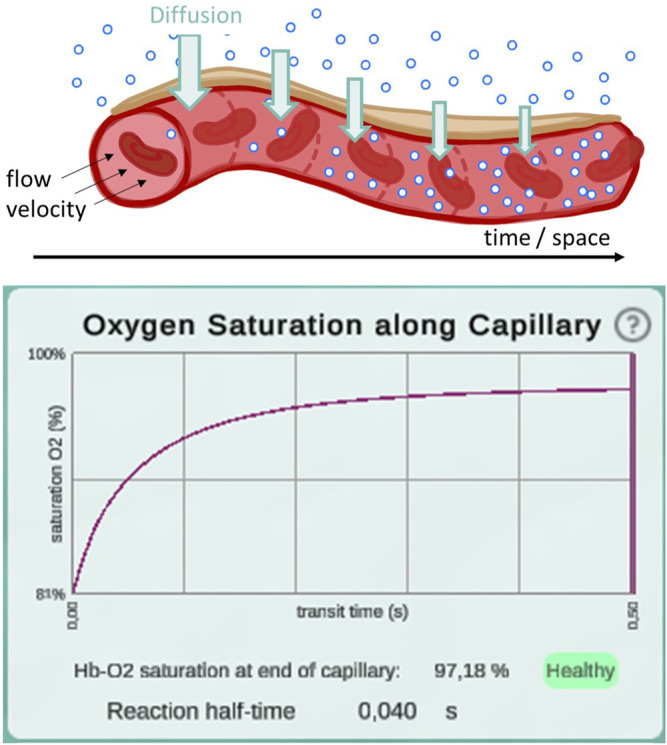
Illustration of the diffusion gradient along the model capillary (top) and a screenshot of the plot displaying oxygen saturation along capillary between 81 and 97% (bottom). This screenshot was taken from a simulation with pO_2_ values of 97 mmHg in the alveolar space and 46 mmHg in the deoxygenated blood. All other parameters remained at their default settings. Reaction half-time is defined as the time point at which 50% of the oxygenation that blood undergoes during its transit along the alveolus is reached.

In a second step, the complete integrative model was validated. We used published experimental data to validate our model. A key contribution of our model is the temporal and spatial resolution. Rather than determining mean values, oxygen partial pressure and saturation gradients along the alveolar capillary are generated. This allows validation of the model in a physiological context. For default parameter settings, 50% of the oxygenation that blood undergoes during its transit along the alveolus is completed after 0.04 s (Figure 3). This measurement was performed for an increase in saturation from 81 to 97%, reaching the reaction half-time at 89%. The corresponding measurement in mice is 0.037 s (Tabuchi et al., 2013) and it has been argued that there are only slight differences between species (Lindstedt, 1984). In summary, we showed that we have correctly adopted and sensibly modified the individual models. Our new integrative model provides results that are consistent with experimental data.

#### 3.1.3 Model Discussion

Our mathematical model was assembled from two existing sub models (Weibel et al., 1993; Dash et al., 2016). One sub model describes the diffusion rate of oxygen from the air into the blood depending on morphological properties (Weibel et al., 1993). In this preceding work, the lung has been defined simplistically as a single container of air and the partial pressure of oxygen in the blood has been considered constant. Some simplifications still exist in our new model. For example, the introduction of a breathing pattern was neglected: Partial pressure changes in alveolar space only occur when respective parameter values are modified by the user (suggests that O_2_ diffusing out of the alveolus is instantly replaced and CO_2_ diffusing into the alveolus is evacuated immediately). Also, blood flow was approximated as a continuous flow of a homogeneous plasma/erythrocyte mixture. However, our new integrative model also features improvements compared to the original models. Instead of steady states, it provides information about oxygen transport over the continuous course of time. It has already been noted that a time-dependent modeling approach is better suited to reconstruct gas exchange in lung tissue than steady-state approaches (Sapoval et al., 2020). Accordingly, the temporal resolution is a valuable improvement to the model.

For validation, we compared reaction half-time results from our model with what has been reported in the literature (Tabuchi et al., 2013). Reaction half-time is defined as the time that elapses until 50% of the oxygenation that blood undergoes during its transit along the alveolus is complete. We measured 40 ms with default parameter settings. Experimentally, a half-time of 37 ms has been determined in mice (Tabuchi et al., 2013). Corresponding theoretical predictions have been slightly lower at 18–32 ms. Tabuchi et al. argue that this discrepancy is due to the fact that the oxygenation process already takes place in the precapillary arterioles, but for the prediction only capillaries were considered. Since only capillaries are considered in *Alvin* as well, we may suspect that our value underestimates the *in vivo* human reaction half-time slightly.

In our model, capillaries are divided into an arbitrary number of sections. The finer grained this discretisation, i.e. the smaller the individual sections and the larger their number, the larger is the resolution of calculated gas dynamics and, thus, the resulting accuracy. However, as described in the following section, our model forms the basis of a visual simulation. With higher resolution, the computational demand grows, especially due to the three-dimensional rendering of the respective capillary sections. Therefore, we manually optimised this detail to maximise the accuracy without jeopardising the simulation’s interactivity.

### 3.2 Visualization and Interactivity: The *Alvin* Application

Interaction with content positively influences its conception (Pike et al., 2009; He et al., 2021) and helps to explore concepts. In parallel with the mathematical model, we developed the *Alvin* simulation software to support the conception and exploration of the gas exchange process in a single alveolus. Addressing the scientific, educational and accessibility requirements (see Methods), we aimed at maximal usability of the software for both research-related and educational applications. Overall, *Alvin* should impart an understanding of the relationship between structure and function of the alveolus.

#### 3.2.1 Visualization

**FIGURE 4:**
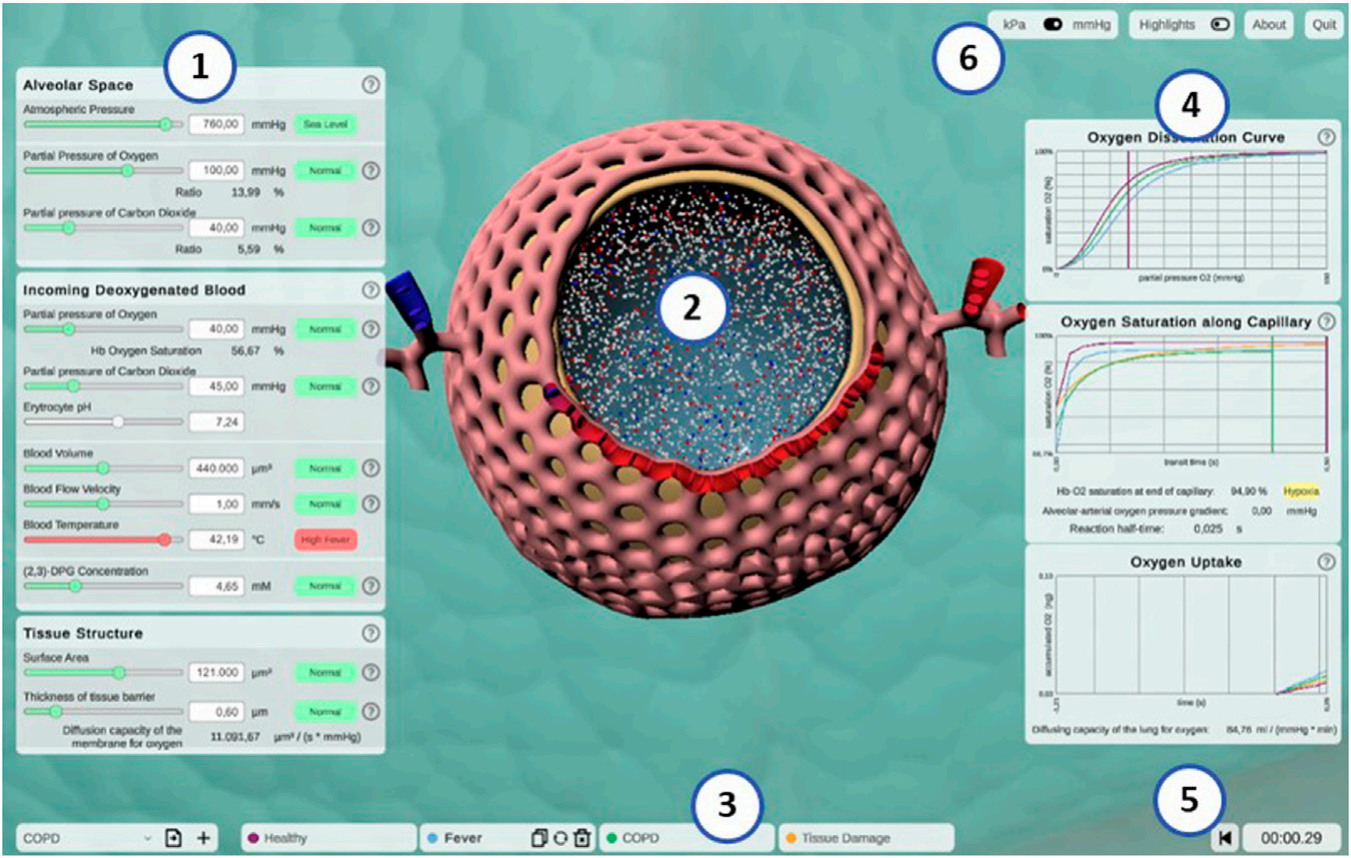
Screenshot of the interactive application *Alvin*. **(1)** Model parameters are grouped in categories and can be configured by the user. Colors and information text provide possible real-world interpretation of the values. **(2)** Animated simulation of an alveolus for the active parameter set provides visualization of the effect of the model parameter values. **(3)** To increase exploratory value, multiple simulation instances can be compared. **(4)** Quantitative simulation output is displayed with plots color-coded for each active instance of the simulation. **(5)** Simulation time is displayed and can be reset. **(6)** Utility functions and settings are available.

*Alvin* is a desktop-based application implemented in Unity. It is available for Windows, macOS and Linux (fulfills (A.1)). The user interface of *Alvin* consists of the following core components: a three-dimensional model of an alveolus illustrating the simulation process, a configuration menu for model parameter values and a panel displaying dynamic graphs (Figure 4) (fulfills A.2.1). A key feature is the ability to run and compare multiple simulation instances at the same time.

The animated, three-dimensional model of an alveolus illustrates the current state of the simulation (Figure 4, center, see also Section S1.3 of the Supplementary Material for further details) (fulfills S.4.1). The alveolus is visually filled with small representations for air molecules, animated to signify Brownian motion. Each one is representing roughly 2 × 10^9^ molecules of oxygen (red spheres), carbon dioxide (blue spheres) or nitrogen (white spheres), respectively. Thickening or thinning of the tissue layer indicates value changes of the model parameter “thickness of tissue barrier”. Erythrocytes are animated and move along the cut-open capillary. The number of erythrocytes proportionally corresponds to a standard value of 5 × 10^6^ cells per μL blood (Pagana et al., 2019). Their relative position on this path is constantly tracked. Oxygen partial pressure (Eq. 1) and hemoglobin oxygen saturation (Eq. 2) gradients are calculated along the same path. This information is combined to color erythrocytes according to their oxygen saturation and to cumulatively total the amount of oxygen taken up by the erythrocytes over the course of the simulation (see Figure 4, graph “oxygen uptake”).

Hence, simulated gas exchange can be retraced by observing the amount of gas spheres crossing the tissue barrier from one side to the other and changes in capillary and erythrocyte coloring (S.4.2). Quantitative outcome of the simulation can be monitored on three different graphs (S.3.1) (Figure 4, right). They show hemoglobin oxygen saturation as a function of pO_2_ in the blood (oxygen dissociation curve) (E.1.2), or of time (oxygen saturation along capillary). Finally, the total amount of oxygen taken up is tracked as a function of the time since the simulation was started or reset. Graphs of different simulation instances are indicated by their respective instance color.

#### 3.2.2 Interactivity

The parameter panel (Figure 4, left) allows users to configure model parameter values. Changes in parameter values yield run-time updates in the 3D visualization and the quantitative graphs (S.2.1). A traffic light color code and keywords provide classification of the chosen parameter values with regard to their healthy or pathological ranges (E.2.2). More information can be obtained by clicking the respective info button (indicated by a question mark) (E.2.1). Model parameters are grouped in terms of the tissue components to which they relate (A.2.2). Visual highlighting in the 3D alveolus model emphasizes these connections (S.4.3). For instance, all tissue components except the capillary are grayed out when the cursor is over the window for model parameters relating to the blood. To examine the process in the 3D model in more detail, it can be moved, rotated or zoomed. Detailed quantitative information can be obtained by hovering over a graph with the mouse. The instance menu allows direct comparison of different parameter settings by running several simulation instances simultaneously (S.2.2) (Figure 4, bottom). Characteristic coloring and custom naming facilitate distinguishing between different simulation instances. A selected instance can be copied, deleted or reset to its initial parameter values. Parameter presets for healthy and common pathogenic conditions are provided (E.1.1) (Table 2). Finally, the user interface contains control elements to monitor or reset simulation time (S.2.3 and S.3.2) and to toggle between pressure units (A.3.2) and visual highlighting modes. More technical details on the implementation of *Alvin* are provided in Section S1.2 of the Supplementary Material. Taken together, these features present interrelationships of the gas exchange process as one explores the system. For example, the user can decrease the alveolar partial pressure of oxygen and observe how this affects the progression of oxygen binding to hemoglobin along the alveolar capillary. One could also observe at what alveolar pO_2_ the blood O_2_ saturation reaches a critically low value at the end of the process. Another example would be to increase the tissue barrier thickness and observe how much the blood oxygen saturation decreases despite unchanged alveolar partial pressures.

**TABLE 2:** Parameter value shifts in presets representing pathogenic conditions. For every condition, pathophysiological issues or symptoms are represented by increased (*↑*) or decreased (*↓*) values of the respective model parameters.

Pathogenic condition	Pathophysiology/Symptom	Parameter value shift
Pneumonia	Fever	Temperature *↑*
	Tissue damage	Surface area *↓*
	Accumulation of fluids and dead cells	Barrier thickness *↑*
ARDS (acute respiratory distress syndrome)	Collapse (alveolar aelectasis)	Surface area *↓↓*
	Fever	Temperature *↑*
COPD (chronic obstructive pulmonary disease)	Impaired exhalation	Alveolar pCO_2_ *↑* and blood pCO_2_ *↑*
	Impaired exhalation	Alveolar pO_2_ *↓*
	Tissue damage	Surface area *↓*
Pulmonary fibrosis	Thickened and scarred connective tissue	Barrier thickness *↑*
	Impaired inhalation	Alveolar pCO_2_ *↓*
Pulmonary embolism	shunt	Blood volume *↓↓*
	shunt	Blood flow velocity *↓↓*

#### 3.2.3 Discussion on Visualization and Interactivity

*Alvin* intends to increase understanding of the complex relationships of gas exchange by highlighting connections and allowing comparison of multiple simulations. Previous interactive systems for gas exchange have pursued a similar goal. (Winkler et al., 1995) have modeled the lung as a complex of abstract gas exchange units (compartments) that can be simulated under individual conditions. (Kapitan, 2008) have created a model of gas exchange that is based on the alveolar gas equation (Sharma et al., 2020) and takes the ratio of ventilation to perfusion into account. Both systems enable simulation of inhomogeneous distribution of ventilation and perfusion. This provides valuable insights into higher-level relationships. In both systems, individual gas exchange units and the whole complex are visualized by means of abstract schematic representations. What happens in detail and how it looks like remains unanswered. *Alvin* fills this gap. The site of gas exchange is no longer abstract—a 3D model illustrates an alveolus in realistic proportions. It conveys the structure of important components (capillary net, tissue barrier). The connection between structure and function is interactively explored in the simulation. Blood flow and tissue thickness in the 3D model adapt to the parameter settings and directly affect the simulation process. What further sets *Alvin* apart from the two systems mentioned above is the possibility of running multiple simulation instances simultaneously. This allows different conditions to be compared directly instead of being modeled and explored one after the other. However, the design of the instance menu in *Alvin* still has a limitation. While qualitative output of several simulation instances can be compared directly, the user is required to switch tabs along the instance menu to compare parameter settings and visual output on the 3D model. This issue should be addressed in future improvements to the system.

The combination of providing parameter value presets as well as allowing parameter configurations by the user enables a presentation of the model that expands existing best-practice (Mogilner et al., 2011). *Alvin* includes a multitude of visualization elements and interaction possibilities. They aim at an intuitive usage of the application and understanding of the gas exchange simulation. It should be assessed whether the use of *Alvin* is actually perceived as intuitive. For this purpose, in the context of a use case study (described in Section 3.3.2), we had a group of users fill out a standardized questionnaire to measure intuitive usability.

### 3.3 Applying *Alvin*: Use Case Studies

We provide two concrete examples for the application of *Alvin*. One of our goals was to ensure that researchers can flexibly explore the model simulation. Here, we demonstrate how the interactive simulation can be used to interpret data from the literature. Second, we report on *Alvin*’s integration into a university level virtual class. The application was used to convey basic and important respiratory processes in the context of a given instructional framework that combined a traditional lecture and instructor based- as well as self-learning.

#### 3.3.1 *Alvin* in Research: Interpreting Data and Testing Predictions

To present a possible use case of *Alvin* for research, we employ the application to check the plausibility of pulmonary diffusion capacity measurements. The pulmonary diffusion capacity (D_LO2_) describes the lungs’ capacity to transport oxygen from the air to the blood. It is defined as the oxygen consumption $\dot{V}O_{2}$ in L/min (oxygen uptake over time) divided by the mean oxygen pressure gradient between alveolar air and capillary blood ΔpO_2_ (Lindstedt, 1984).

$$D_{{\text{L}}_{{\text{O}}_{2}}}=\frac{\dot{V}O_{2}}{{\Delta pO}_{2}}$$ (3)

**FIGURE 5:**
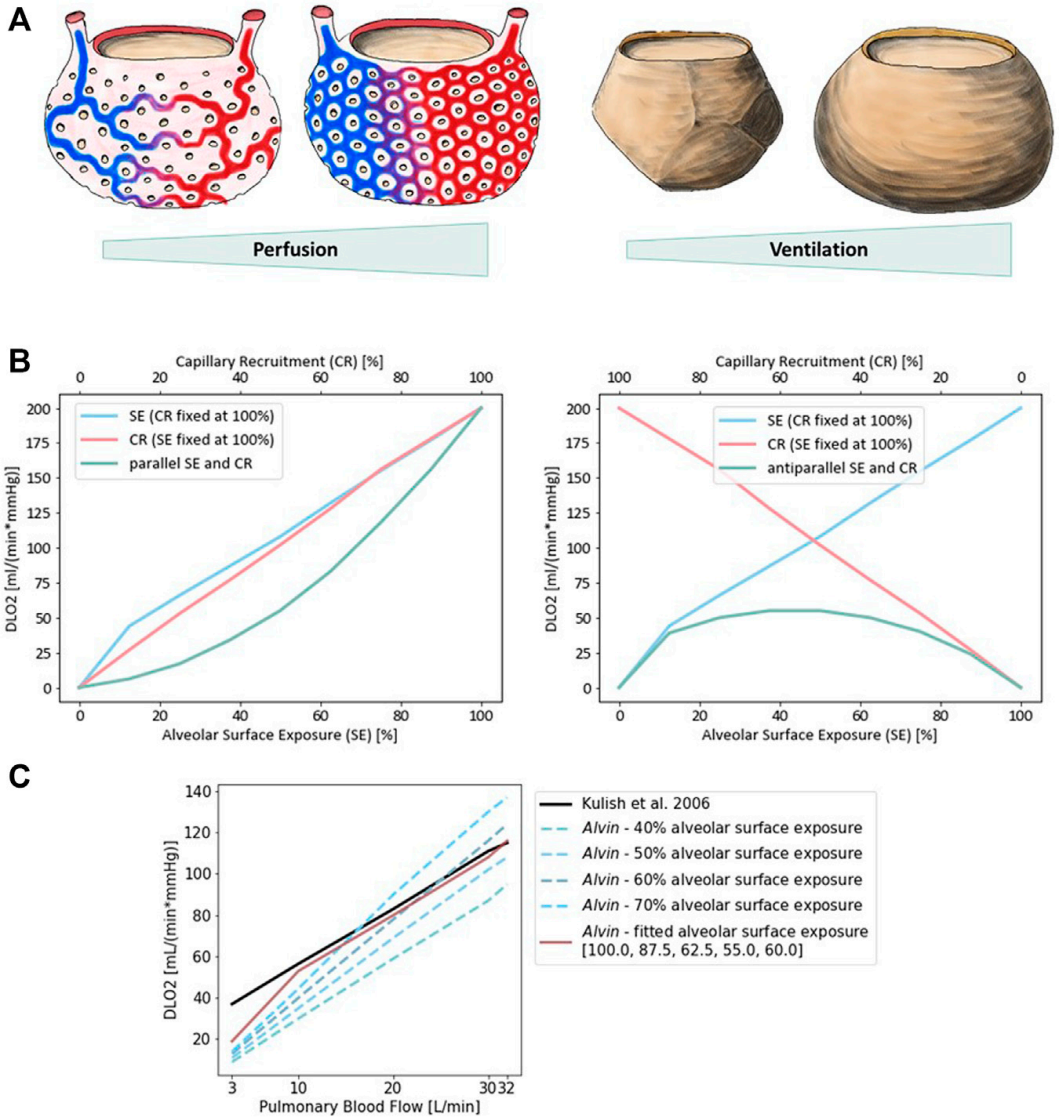
Diffusion capacity of the lung for oxygen (D_LO2_) strongly depends on perfusion and ventilation. **(A)** Illustration of capillary recruitment (left) and alveolar expansion (right). **(B)** Diffusion capacity of the lung for oxygen (D_LO2_) depending on capillary recruitment and alveolar expansion for a parallel (left) and antiparallel combination (right). Alveolar expansion and the ensuing surface exposure are simulated in *Alvin* by increasing alveolar surface area from 0 (0%) to 207,000 μm^2^ (100%) in steps of 12.5%. Capillary recruitment is represented by capillary blood volume increase from 0 (0%) to 808,000 μm^3^ (100%) in steps of 12.5% in *Alvin*. **(C)** Comparison to published D_LO2_ estimates (Kulish, 2006) (black). Pulmonary blood flow was interpreted as blood volume in *Alvin*, assuming a flow velocity of 1.5 mm/s and morphological features (mean capillary length of 500 μm (Weibel et al., 1993) and maximum volume of alveolar capillary bed 808,000 μm^3^ (Gehr et al., 1978; Ochs et al., 2004)). Alveolar surface exposure was fixed at constant values (blue dashed lines) and adjusted with increasing pulmonary blood flow (red line).

Physiological estimates of D_LO2_ are usually derived from measurements of diffusion capacity for carbon monoxide (D_LCO_) (Forster, 1964; Crapo and Crapo, 1983). Normal values of D_LO2_ at rest are around 30 ml/(mmHg × min) (Hsia et al., 2016). Determination of D_LO2_ based on morphometric data has resulted in a value of 158 ml/(mmHg × min) (Weibel, 2009) and thereby exceeds physiological approximations considerably. There are several reasons for this discrepancy (Hsia et al., 2016). One of them is that for the morphological estimation, a complete perfusion of the capillaries is assumed and the entire alveolar surface is included in the calculations (Weibel, 1970). Under normal conditions, only about 50% of capillary segments in the alveolar wall are perfused by erythrocytes and thus contribute to gas exchange (Okada et al., 1992) (Figure 5A). Increasing blood pressure (e.g., due to increased cardiac output) leads to recruitment of further capillary segments. In the perfusion fixed, air-filled lung, only about 60–70% of the alveolar surface area is exposed to air (Gil et al., 1979; Bachofen et al., 1987). In addition, lung volume changes during respiration depending on the transpulmonary pressure. It has been proposed that alveolar recruitment may be responsible for these volume changes, i.e., opening and closing of alveoli (Carney et al., 1999). However, *in situ* studies rather suggest an increase in alveolar size (D’Angelo, 1972). In terms of the model parameters in *Alvin*, both hypotheses manifest themselves in changes in the alveolar surface area available for gas exchange. A surface area of 207,000 μm^2^, measured in inflation-fixed lung tissue (Stone et al., 1992), describes a maximum surface exposure of 100%. The default surface area setting in *Alvin* is 121,000 μm^2^ and thus corresponds to an exposure of 58%. This value was taken from a study in which the tissue was perfusion fixed (Mercer et al., 1994). Capillary recruitment in *Alvin* is reflected in capillary blood volume, for which the default value 404,000 μm^3^ represents 50% recruitment. By mimicking the ratios of capillary recruitment and alveolar surface area in *Alvin*, one can directly trace the effect on D_LO2_. 100% alveolar surface exposure and 100% capillary recruitment in *Alvin* yield a D_LO2_ of 200 ml/(mmHg × min). 58% alveolar surface exposure and 50% capillary recruitment result in a D_LO2_ of 61 ml/(mmHg × min).

Alveolar surface area and capillary recruitment impact D_LO2_ estimates almost linearly (Figure 5B). Additionally, it is interesting to observe their synergistic effect, as ventilation and perfusion are regulated to match (reviewed in (Wagner, 1981; Petersson and Glenny, 2014)). Parallel increase of both alveolar surface exposure and capillary recruitment lead to a non-linear increase in D_LO2_, slowly at first and then more rapidly. Consistently, anti-parallel combination of these factors yields generally low D_LO2_ estimates, with a peak at 50% each. Quantification of this relationship in *Alvin* can be used to interpret other data from the literature. For instance, D_LO2_ has been estimated from measurements of D_LCO_ and pulmonary blood flow (Kulish, 2006). To recreate these estimates, pulmonary blood flow, expressed in volume per unit time, was interpreted as alveolar blood volume in *Alvin*. Assuming a constant blood flow velocity of 1.5 mm/s, the alveolar blood volume was obtained from the mean capillary length of 500 μm (Weibel et al., 1993) and the maximum volume of alveolar capillary bed 808,000 μm^3^ (Ochs et al., 2004; Gehr et al., 1978). Under these conditions, D_LO2_ was determined in *Alvin* with varying alveolar surface area settings (Figure 5C). The resulting D_LO2_ graphs all differed in slope from the published data (Kulish, 2006). Thus, Kulish’s predictions did not appear to have been based on constant alveolar surface exposure. By adjusting alveolar surface area values (100, 87.5, 62.5, 55.0 and 60% surface exposure) along with increasing blood flow (3, 10, 20, 30 and 32 L/min), the results could finally be reconstructed. This fitting was not successful at very low blood flow values.

This is only one example of how to employ *Alvin* to investigate correlations in a broader sense or to reproduce data from the literature to gain further insight. Further questions could address the kinetics of gas exchange. One possibility would be to investigate the threshold conditions under which the blood is still sufficiently oxygenated within the transit time.

#### 3.3.2 *Alvin* in Higher Education: Physiology Lab Course

For application in teaching, the benefits of an interactive simulation have been perceived and exploited since the 1980s (Dewhurst et al., 1988; Davis and Mark, 1990) and are still being pursued today (Jacob et al., 2012; Tworek et al., 2013). Therefore, we integrated *Alvin* into a university level class on human biology, specifically an online practical session on blood and respiration. *Alvin* was used to support the online session by providing an interactive model of the cooperation of the bloodstream and the respiratory system. The suitability of *Alvin* for this course was measured with an online questionnaire.

The course was scheduled for 2 h and 45 min. The participants consisted of students of teaching Biology, specifically of the German levels of *Grundschule* (elementary school/grades 1–4, mostly third year students), *Mittelschule* (secondary school/grades 5–8, mostly third year students) and *Gymnasium* (grammar school/grades 5–13, mostly fifth year students). After an introduction into the topic “Blood and Respiration” in the form of a 45 min lecture, *Alvin* was presented briefly, explaining how to use the application and interpret the 3D model and graphs. Participants were given a few minutes to familiarize themselves with *Alvin*. They were then asked for feedback as they worked with the application. An online questionnaire was provided to collect responses. Participation was voluntary and could be withdrawn throughout the event. Submitting the questionnaire as a whole, or answering individual questions, was not mandatory. The questionnaire was split in four parts. The entire questionnaire, translated from German, can be found in the Supplementary Material (Supplementary Section S2.1).

**FIGURE 6:**
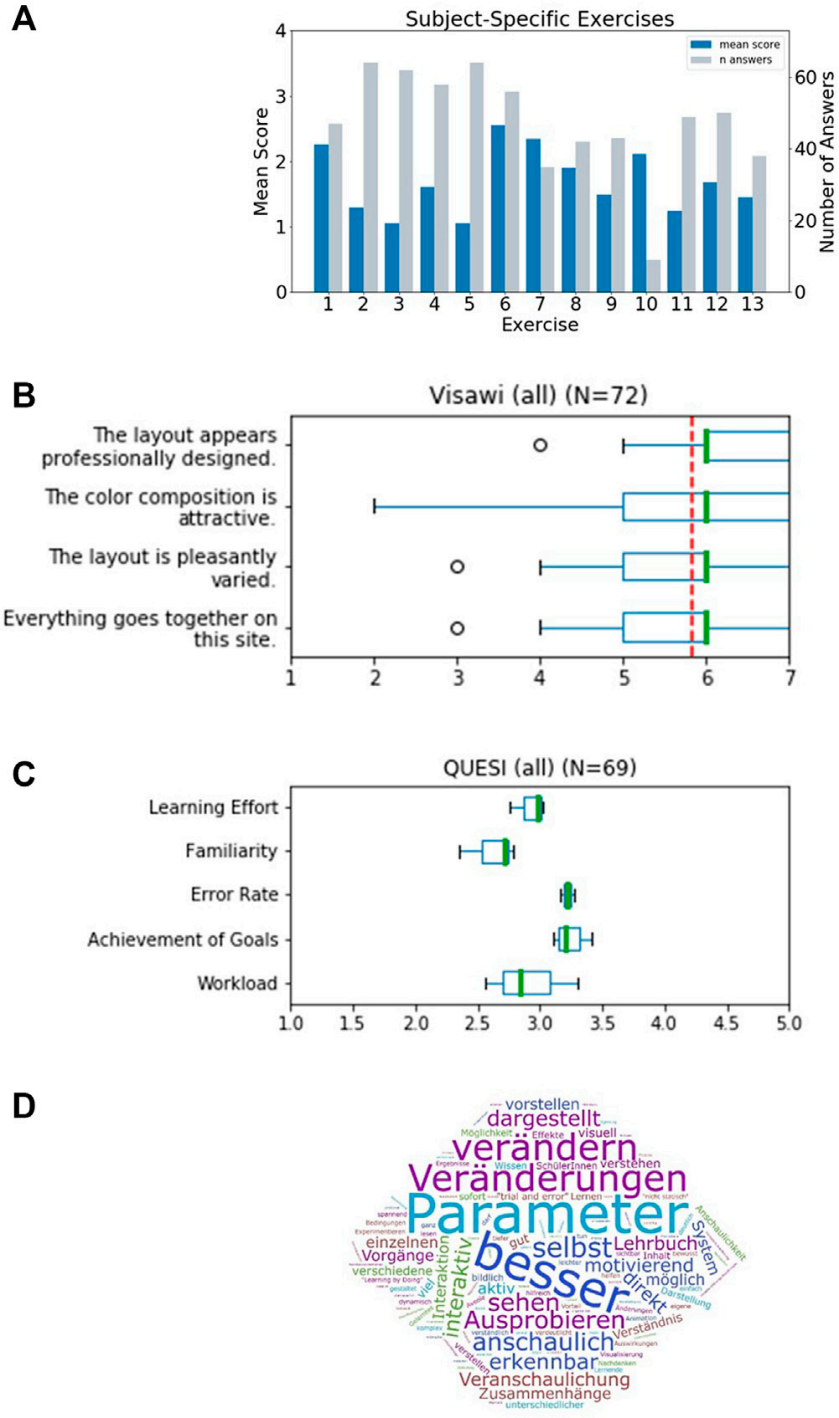
Results of a survey for undergraduate students that worked with *Alvin* in a physiology lab course. **(A)** Evaluation of thirteen subject-specific exercises. Responses were scored 1 - correct, 2 - partially correct (e.g., subsequent faults), 3 - unclear to 4 - incorrect. The mean score for every exercise was determined. The individual exercises were answered by different numbers of participants (grey bars). **(B)** The standardized Visawi-s survey (Moshagen and Thielsch, 2021) addresses design features. The 72 participants rated from 1 (strongly disagree) to 7 (strongly agree). The mean score over all four categories was 5.8 (red, dashed line). **(C)** Results on usability from the standardized survey QUESI (Hurtienne and Naumann, 2010). Five subscales are assessed, with higher scores obtained the more intuitive the use of the system was perceived to be. The mean overall QUESI score from 69 forms was 2.98. **(D)** Participants were asked “which benefits do you see in this system compared to a traditional text book?“. A frequency analysis on the answers was performed. The most recurrent terms were (translated from German): “parameter”, “better”, “modifiy”, “changes”, “by oneself”, “illustrative”, “testing”, “see”, “illustrated”, “apparent”, “interactive” and “immediate”.

The first part consisted of a generic demographic questionnaire, extended by specific questions to assess the formal background of the students and their experience with the subject. We received *N* = 73 valid submissions which were at least partially answered. Of the *N* = 73 surveys received, 11 self-identified as male, 56 as female. The participants all had some prior knowledge of respiratory physiology acquired in a physiology lecture in the previous semester and/or in school or training. In this lecture, basics about the structure and physiology of the lungs as well as the functions of the blood as a transporter of respiratory gases were explained. About half of the group (*N* = 34) could be assumed to have even deeper prior knowledge, as they stated that they had studied further literature in addition to the lecture in question. Participants could be divided into groups with prior knowledge level 1 and 2 accordingly. None of the participants reported being affected by color blindness. The second part contained 13 different exercises addressing respiratory processes in the alveolus. These exercises provided instructions on how to integrate *Alvin* into solution approaches. Among other things, these exercises highlighted well-known relationships and phenomena such as the Bohr effect (Riggs, 1988). Responses were rated on a scale of 1–4 (with 1 indicating perfect answers). The individual exercises were answered by different numbers of participants (Figure 6A). Exercise 7 and 10 were answered by less than half of the participants and were therefore not included in the mean overall score of 1.6. Participants with prior knowledge of level 1 performed similarly well to participants with prior knowledge of level 2 (Supplementary Figure S1).

The third part consisted of two standardized questionnaires to assess the visual aesthetics and the usability of the application: Visawi-s (Visual Aesthetics of Websites Inventory- short version) (Moshagen and Thielsch, 2021) and QUESI (Questionnaire for Measuring the Subjective Consequences of Intuitive Use) (Hurtienne and Naumann, 2010). Visawi-s (Moshagen and Thielsch, 2021) captures four central aspects of aesthetics from the user’s perspective: simplicity, diversity, colorfulness and craftsmanship. Participants were presented with statements targeting these four aspects. They rated them on a scale from 1 (strongly disagree) to 7 (strongly agree). The mean overall (*N* = 72) Visawi-s score was 5.8 (see Figure 6B). The standardized QUESI provided a measure of usability (Hurtienne and Naumann, 2010). It is based on the assumption that intuitive use is the unconscious application of prior knowledge leading to effective interaction. It can be divided into the following subscales: Subjective mental workload, perceived achievement of goals, perceived effort of learning, familiarity, and perceived error rate. The total score of the questionnaire is equal to the mean across all five subscales. Generally, higher scores represent a higher probability of intuitive use. Participants’ (*N* = 69) assessments of the use of *Alvin* resulted in a QUESI score of 2.98 (Figure 6C). Published benchmark values for mobile devices and applications (Naumann and Hurtienne, 2010) range from 2.39 (Alcatel One Touch 311) to 4.23 (Nintendo Wii). Familiar products generally perform better in the QUESI (Naumann and Hurtienne, 2010). Hence, participants’ prior experience with similar systems in a broader sense, for example, with computer games in general, is important. The majority of our participants (*N* = 59) reported rarely (yearly to never) playing computer games. The minority (*N* = 29) reported using computer games frequently (monthly to daily).

Finally, the questionnaire included customized questions on the use of *Alvin* (evaluation can be found in Supplementary Section S2.2) and free-form questions aimed at the acceptance of the software in the educational context. One of them was “Which benefits do you see in this system compared to a traditional text book?”. A frequency analysis on answers revealed the highest recurrence for the terms “parameter”, “better”, “modifiy”, “changes”, “by oneself”, “illustrative”, “testing”, “see”, “illustrated”, “apparent”, “interactive” and “immediate” (Figure 6D). A question asking for general feedback was responded to in part with constructive criticism. In particular, it was noted that the content of *Alvin* and the subject-specific tasks were too complex for this introductory event. Or that more time would have been necessary to familiarize oneself with the application. In addition, some reported problems switching between the German lecture content and the English-language application. The participants solved the subject-specific exercises for the most part correctly. It can thus be concluded that *Alvin* is suitable to assist in solving such tasks. Responses to free-text questions suggest which aspects of working with *Alvin* stood out as particularly positive. These include the possibility to interact with the simulation by configuring model parameters and the freedom to independently test different conditions. It was also perceived positively that the simulated processes are presented very illustratively in *Alvin*.

#### 3.3.3 Discussion of Use Cases

Our exemplary use cases show the applicability of *Alvin* in research and in education. We showed an investigation of the dependencies of D_LO2_ on surface area and blood flow in *Alvin*. Physiological estimates often only consider information about blood flow (Kulish, 2006). By reproducing these estimates in *Alvin*, one can draw conclusions about the alveolar surface. At particularly low blood flow values, it is not possible to reproduce the physiological estimates for D_LO2_ in *Alvin*. This could have different causes. In the logic of the model and the definition of D_LO2_, it is ensured that D_LO2_ is zero when the blood volume is zero. The physiological estimates in (Kulish, 2006) do not seem to meet this criterion. (note: One cannot be certain, however, because in Kulish et al. (Kulish, 2006) the lowest reported value for blood flow is 3 L/min). It is possible that our model does not produce reliable results in the range of low blood volume values. Another possibility is that the derivation of D_LO2_ from D_LCO_ is not reliable in low ranges. This plausibility check shows how *Alvin* can be used to support or challenge published data. Drawing on known relationships, additional information can be obtained from previous results.

We also showed that *Alvin* is helpful for communicating respiratory processes in the training of undergraduate students. Well-known processes or phenomena like the Bohr-Effect (Riggs, 1988) can be recreated in *Alvin* and compared with results reported in the literature. Interactivity of the simulation enables experimentation with the model and exploration of its limitations. This aspect was also positively highlighted by participants of the physiology lab course in free-form answers of our questionnaire. The results of the QUESI and VISAWI questionnaires on their own do not allow for quantitative conclusions on usability or aesthetics of the application. This would require comparing them to corresponding results from comparable test situations (for example, about similar systems). At this point, one can only state that the replies did not hint at unknown issues. Instead, they were aligned with our expectations that participants should be able to operate the system autonomously and find its use appealing and relatively intuitive.

In summary, the integration of *Alvin* into physiology classes at the university level was successful. Beyond that, issues were pointed out where the implementation could be optimized in the future. Prominent and consistent were requests for more time to engage with *Alvin*. We deliberately refrained from providing the application to the participants in advance of this course to avoid a mutual influence of the participants regarding their experience with *Alvin*. This was important for the evaluation with the standardized questionnaires. For general use in teaching, however, this does not have to be taken into account. On the contrary, an exchange between students about the system could increase its learning value. We conclude that *Alvin* is less suitable to be included in a single physiology lesson. Instead, we recommend that students be made aware of the app ahead of time or to invest several course sessions.

## 4 Conclusion and Outlook

Interactive, visual simulations allow communicating modeling results and thereby help to further our understanding of the process under study. We presented *Alvin*, an application for simulating gas exchange in a single alveolus. The simulation is based on a mathematical model for the entire transport process of oxygen from the air to hemoglobin of the blood. We claim that having the goal of an interactive, visual simulation in mind when developing a mathematical model is beneficial for the modeling process. It resulted in a specific requirement for the model: In order to be able to map the course of the simulation on a three-dimensional tissue model, it had to be temporally and spatially resolved. Models evolve by being revised and improved over and over again (Drubin and Oster, 2010). If one assumes that a model can be better developed the more experts review it, then it is advantageous to make the model freely and intuitively accessible.

We argue that interactive visualization offers an engaging way to communicate theoretical models to other scientists and students. When cooperating with experimenters, it is important for theorists to present their models in the most accessible way possible. This creates as large a basis for discussion as possible in order to jointly plan further experiments or model refinements. By making model parameters intuitively configurable, any experimenter can compare his or her own measurements with the modeling results. By including undergraduate students in the target group for *Alvin*, we ensured that only a minimum of prior knowledge is required for its usage.

In the future, we plan to extend our model to encompass a system of multiple alveoli and their associated vessels. This will allow us to address further questions and complex relationships regarding gas exchange in lung tissue. It is known that the ventilation-perfusion relationship, and therefore the diffusion-perfusion relationship, has a strong influence on D_LO2_ (Hyde et al., 1967; Hammond and Hempleman, 1987). An evolution of *Alvin* that includes an alveolar sac or a whole acinus with differently ventilated and perfused alveoli can provide valuable insights. This could also be used, for example, to further investigate the hypothesis of precapillary oxygen uptake (Tabuchi et al., 2013). It states that the oxygenation process already takes place in the precapillary arterioles before the blood reaches the alveolar capillary bed.

Rather than just presenting the data that results from a newly developed model, it is worthwhile to implement the model in a way that allows for interaction. Visualizing the simulation makes the engagement with the model more intuitive and accessible to a broader target group. Empiricists and theorists look at a system from different angles. Some work in a bottom-up fashion and take local samples and draw conclusions for the overall system. Others create abstract models for the overall system top-down and try to approach the truth by introducing more and more details. Only by working closely together can these two perspectives efficiently contribute to reliable results and become a “middle-out” approach (Noble, 2008). The communication of the achieved findings or predictions plays an important role here. We contend that interactive, visual simulations of theoretical models, as we have implemented with *Alvin* on respiratory processes in the alveolus, will make an important contribution to bridging the gap between empiricists and theorists.

## Data Availability

The original contributions presented in the study are included in the article/Supplementary Material, further inquiries can be directed to the corresponding author. We provide the source code of *Alvin* at https://github.com/scfischer/schmid-et-al-2022.
