# Supplementary material for: Interactive, Visual Simulation of a Spatio-Temporal Model of Gas Exchange in the Human Alveolus
Source: Front Bioinform. 2022 Jan 26;1:774300. doi: 10.3389/fbinf.2021.774300 (PMC9580865; doi:10.3389/fbinf.2021.774300)
Supplement: Supplementary file 1 [file DataSheet1.pdf]

Supplementary Material

S1 METHODS

S1.1 Requirements Analysis

Our interdisciplinary team developed concepts to achieve our goals. In a user-centered engineering approach, a set of requirements was defined:

Scientific Requirements

S.1 Gas exchange model suitable for interactive configuration.
  S.1.1 Model parameters as public variables.
S.2 Interfaces for interaction.
  S.2.1 UI that allows configuration of model parameters and a simulation that responds directly to these configurations.
  S.2.2 The application should allow comparisons of simulations with different model configurations.
  S.2.3 Possibility to reset the simulation.
S.3 Quantitative simulation output.
  S.3.1 Quantitative results in the form of graphs and key conclusion values.
    S.3.1.1 Dynamic plots that provide immediate feedback to model configurations by the user.
    S.3.1.2 Readouts that allow comparison with experimental data (for model validation and application in research): DMO_2, $D_{L_{O_2}}$, reaction half-time.
  S.3.2 Possibility to follow the simulation time.
S.4 Visual feedback that emphasizes the connection between structure and function of the alveolus.
  S.4.1 Zoomed-in system visualization: realistic, three-dimensional model of an alveolus.
  S.4.2 Illustration of both model configurations (starting partial pressures of O_2/CO_2, blood volume and -flow velocity, thickness of tissue barrier) and simulation output (partial pressures of O_2, erythrocyte O_2 saturation) on the 3D model.
  S.4.3 Emphasis on the connection between parameter configurations and output.

Educational Requirements

E.1 Presentation of educationally relevant respiratory phenomena.
  E.1.1 Consideration of both healthy and common disease conditions (pneumonia, ARDS, COPD, pulmonary fibrosis, pulmonary embolism) and means of comparison.
  E.1.2 Consideration of the Bohr/Haldane effect.
    E.1.2.1 The model parameters to be configured must include those that are decisive for the Bohr effect.
    E.1.2.2 Oxygen binding curve as an additional quantitative output.
E.2 Facilitate autonomous work with the application.
  E.2.1 Background information on model parameters, disease conditions and output in the form of explanatory text.
  E.2.2 Helping the user understand how to relate the simulation to physiology
    E.2.2.1 Classification of the parameter configuration in relation to physiological value ranges.

E.2.2.2 Classification of the (quantitative) simulation output in relation to physiological value ranges.

Accessibility Requirements

A.1 Compatibility with common devices (computers or tablets with windows, iOS or linux).
A.2 Simple and clear GUI (to enhance the intuitive use of the system).
  A.2.1 A GUI that juxtaposes model configuration and output (visual and quantitative) to provide an overview of the most important functionalities at first glance.
  A.2.2 Well arranged design of parameter menu.
    A.2.2.1 Model parameters sorted into meaningful groups.
    A.2.2.2 Sliders (convenient) and input fields (explicit) for parameter configuration.
  A.2.3 Detailed information on model parameters and output that appears only when needed (in the form of pop-up windows and tooltips) to avoid overloading the GUI.
A.3 Applicability to the widest possible range of scientific issues.
  A.3.1 Possibility to configure a wide range of parameter values.
  A.3.2 Common gas units (mmHg and SI unit kPa).

S1.2 Implementation of *Alvin*

The application *Alvin* was built in Unity version 2020.1.16f1 (`https://unity.com/`). Unity is a platform for creating interactive real-time content. The mathematical model (see Section 3.1.1) provides the basis for the simulations. Parameter value changes in *Alvin* result in instantaneous updates of the visual output. All calculations are performed in the pressure unit mmHg. For the purpose of visualization, simulation time is slowed down by a factor of 40 compared to the gas exchange process *in vivo*.

S1.3 Three-dimensional visual model of an alveolus

The three-dimensional, mesh-based geometric model of an alveolus was created in Blender® version 2.82 (`https://www.blender.org/`). The model resembles a real alveolus not only in its general appearance, but also in proportions. The tissue barrier, consisting of alveolar lining fluid, epithelial cells and connective tissue fibers are represented by a single layer in this model. This layer forms a truncated sphere with diameter, volume and surface area comparable to the corresponding values of an alveolus reported in the literature (Table S1). A network of hollow channels was modeled around this tissue layer, representing the capillary network enveloping the alveolus. The properties of the model network are chosen such that the relative magnitude of the volume and the surface area, as well as the relative radius and the length of the individual segments agree with the respective measured values from the literature (Table S1). From these morphometric features, a mean number of 52 capillaries surrounding an alveolus was derived (Table 1). As the alveolar capillary network has no distinct capillaries, this value serves as a rough reference value. To determine the number of capillaries in our model, the network could be interpreted as parallel capillaries with cross-links. Our model contains 41 'parallel capillaries'. The cross-links between them contribute to gas exchange as well. To facilitate visualisation of the blood flow, a capillary was cut open longitudinally, thereby exposing the inside of the channel. In addition, the inflow and outflow of blood is indicated with the help of additional capillaries that connect to the capillary net.

S1.4 Simulation Output

In the graph panel, dynamic plots record the course of the simulation quantitatively. The plot “oxygen saturation along capillary” presents results obtained directly from the simulation calculations. The oxygen uptake, presented in another graph, is calculated assuming a standard amount of $270 \cdot 10^6$ hemoglobin molecules per erythrocyte (Pierigè et al., 2008). Considering the parameter values from the configuration menu, but independent of the rest of the simulation, the “oxygen dissociation curve” is calculated for a range of partial pressure values of oxygen.

If several simulation instances are active at the same time, the respective results are displayed together in the graphs, while only information about the selected instance is considered in the 3D visualisation in the center.

Two different prototypes of *Alvin* were implemented for the different use cases described in this work. One prototype was adapted for educational use (Section 3.3.2) such that the application has two levels of different complexity. In the first level, only one simulation instance can run at a time. The second level with full complexity is unlocked by an access code. The other prototype features additional readouts. ”Membrane” diffusing capacity for oxygen DMO_2 and reaction half-time were required for model validation (Section 3.1.2). For the application example in research (Section 3.3.1), the output for diffusion capacity of the lung for oxygen was added. This second prototype is depicted in Figure 4 and available for download at `https://go.uniwue.de/alvin`.

S2 CASE STUDY: INTEGRATION OF *ALVIN* IN A UNIVERSITY LEVEL PHYSIOLOGY LAB COURSE

S2.1 Questionnaire

The questionnaire was translated from the German original.

Demographics

In this section, we ask you to answer questions for general demographic information. These are relevant for a correct interpretation of your further answers.

1. Please indicate your age.
2. Please indicate your biological sex.
3. Do you have a visual impairment and will it be compensated for while using the system?
4. Are you affected by color vision deficiency or color blindness?
5. What handedness do you have?
6. On average, how often do you use the following media?
    - Internet
    - Computer (in general)
    - Computer games
    - Smartphone
    - Tablet
7. How would you rate your fluency in German?

In this section, we ask you to answer questions about your prior knowledge in the subject area of the course and regarding your previous educational background.

8. In the context of which study program are you attending this event?
9. What semester are you in?
10. Did you attend the Human Biology lecture in the summer semester of 2020?
11. Have you studied the literature recommended in the above lecture on the subject of respiration?
    - N.A. Campbell and J.B. Reece. *Biology*. Always learning. Pearson Deutschland, 2015. ISBN: 9783868942590.
    - Robert F. Schmidt, Florian Lang, and Manfred Heckmann. *Physiologie des Menschen*. Springer-Lehrbuch. Springer-Verlag Berlin Heidelberg, 2011. ISBN: 978-3-642-01651-6.
12. Do you have other relevant prior knowledge from other sources?
    - School
    - Apprenticeship
    - Personal initiative

Subject-specific exercises

In this group of questions, you will be given tasks that you can answer using the system. We ask you to discuss comments on the use of the app only in a joint round at the end of the event in order to minimize influencing the other participants. Now, familiarize yourself with the application. Look at how the graphs change in response to the controllers. Also observe how different disease patterns affect the values.

1. Which correlations between the course of the oxygen saturation curve (”Oxygen saturation along capillary”) and the visualized simulation can you identify?

In this and the following blocks of questions, you will be given tasks to answer using the system. After each task (there are 3 tasks in total, each with subtasks), the answers will be discussed in plenary. We ask you to discuss comments on the use of the application only in a joint round at the end of the event in order to minimize influencing the other participants.

2. How does the oxygen dissociation curve change, when the body temperature rises to 40 °C (fever)?
3. How does this affect the ability of hemoglobin to bind oxygen in the lungs?
4. How does it affect the ability of hemoglobin to deliver oxygen to tissues?

Fever is normally accompanied by an increase in respiratory rate. By increasing the respiratory rate, the increased CO_2 produced by the increased metabolism during fever can be better exhaled. The partial pressure of CO_2 in the blood affects the ability of hemoglobin to bind O_2.

5. By how many mmHg must the venous CO_2 partial pressure be lowered to achieve the same oxygen saturation at 40°C as at 37°?

The cruising altitude of passenger aircrafts is around 10 to 13 km. At this altitude, the partial pressure of oxygen is only between 30 and 44 mmHg. Therefore, the air pressure in the cabins of passenger aircrafts is artificially increased, but only to a level corresponding to the air pressure at about 2000-2500 m above sea level. Thus, an oxygen partial pressure of approx. 60 mmHg is achieved in venous blood.

6. What oxygen saturation does this correspond to?
7. At what alveolar pO_2 can a healthy person achieve this?
8. What oxygen saturation does the blood of a patient suffering from COPD reach at the same atmospheric pressure?
9. What happens to the oxygen saturation of a person suffering from COPD if he or she develops a fever during a flight?

In this block of questions, you will be given tasks to answer using the system. Please configure the application using the activation code provided in the lecture. We ask that you do not discuss comments on the use of the application until a joint round at the end of the course to minimize influencing the other participants.

10. Sometimes a lung has to be surgically removed due to a disease. What effects does this have on the oxygen saturation of the blood?

In tissues with very high metabolic rates, for example heavily used muscles, the CO_2 concentration can increase.

11. How does this affect the oxygen dissociation curve?
12. How does it affect oxygen uptake in the lungs and oxygen delivery in the tissues?

Start two simulation instances with the parameters for a healthy person. Increase the partial pressure of CO_2 in the arterial blood of one instance to 75 mmHg. Using the oxygen dissociation curves, measure the absorbed oxygen in the lungs and the delivered oxygen in the tissues.

13. At which blood pCO_2 is more oxygen available to the tissue? This phenomenon is called the Bohr effect.

Note: Exercises 14 to 17 were not covered in the educational case study (Section 3.3.2) due to time constraints.

Athletes, especially high-altitude mountaineers, can adapt to conditions at high altitude by training for longer periods at low oxygen partial pressure. This increases the diphosphoglycerate (DPG) concentration in the erythrocytes. Now, we would like to understand why this is beneficial. Start a simulation instance with the parameters for a healthy subject. First, reconstruct the conditions that exist when climbing at high altitudes: Decrease atmospheric pressure until alveolar pO_2 drops to a low value such as 40 mmHg. Arterial pO_2 is also reduced in these conditions. Set this to 30 mmHg.

14. What is the oxygen saturation of the blood?

Duplicate the instance. Now, set the DPG concentration in one of the two instances to the maximum value (adjustment to high altitude).

15. What happens to the oxygen dissociation curve?
16. What happens to the oxygen saturation of the blood?
17. The effect you observed initially appears to be rather disadvantageous. Now, measure the oxygen saturation in the lungs and in the tissue in the respective graphs and determine the difference between these values.

The questions will now be discussed in the plenum of the event. We ask you to discuss comments on the use of the application only in a joint round at the end of the event to minimize influencing the other participants.

The phase of active use of the system is now complete. In the following, we ask you to answer questions about your user experience. This is for systematic evaluation of the system. Please note that for the first two questions a ”soft” inquiry will appear if you do not answer them or answer them only partially. The corresponding groups of questions are standardized questionnaires, where a complete answer has a lot of value. Of course, you can still skip them unanswered if you wish.

QUESI – Questionnaire for Measuring the Subjective Consequences of Intuitive Use

This is a standardized questionnare (Hurtienne and Naumann, 2010). Try to base your assessment of the system solely on the use of the system (and not, for example, on the difficulty of the task itself). There are no right or wrong answers. Please answer spontaneously and do not omit any questions.

Answer scale with equidistant levels: 1 = ”Fully disagree”, 2 = ”Mainly disagree”, 3 = ”Neutral”, 4 = ”Mainly agree”, 5 = ”Fully agree”.

1. I could use the system without thinking about it.
2. I achieved what I wanted to achieve with the system.
3. The way the system worked was immediately clear to me.
4. I could interact with the system in a way that seemed familiar to me.
5. No problems occurred when I used the system.
6. The system was not complicated to use.
7. I was able to achieve my goals in the way I had imagined to.
8. The system was easy to use from the start.
9. It was always clear to me what I had to do to use the system.
10. The process of using the system went smoothly.
11. I barely had to concentrate on using the system.
12. The system helped me to completely achieve my goals.
13. How the system is used was clear to me straight away.
14. I automatically did the right thing to achieve my goals.

Visawi-s - Visual Aesthetics of Websites Inventory- short version

This is a standardized questionnare (Moshagen and Thielsch, 2021). On a scale of 1 (strongly disagree) to 7 (strongly agree), please rate the extent to which you agree with the following statements regarding the system.

1. The layout appears too dense. (r)
2. The layout is pleasantly varied.
3. The color composition is attractive.
4. The layout appears professionally designed.
5. The layout is easy to grasp.
6. The layout is inventive.
7. The colors do not match. (r)
8. The layout is not up-to-date. (r)

9. Everything goes together on this site.
10. The design appears uninspired. (r)
11. The choice of colors is botched. (r)
12. The site is designed with care.
13. The site appears patchy. (r)
14. The layout appears dynamic.
15. The colors are appealing.
16. The design of the site lacks a concept. (r)
17. The layout appears well structured.
18. The design is uninteresting. (r)

Negatively-keyed items are indicated by (r) and are reverse-scored.

Customized questions on the use of *Alvin*

1. On a scale of 1 (strongly disagree) to 7 (strongly agree), please rate the extent to which you agree with the following statements regarding the system.
    - I frequently changed the ”Incoming Deoxygenated Blood” parameter values for completing the tasks.
    - I frequently changed the ”Alveolar Space” parameter values for completing the tasks.
    - I frequently changed the ”Tissue Structure” parameter values for completing the tasks.
    - The system supported me in the configuration and interpretation of the parameters.
    - I was confused by the information provided by the system.
    - Assessment of the parameter values was useful for my understanding of the processes.
    - I found the ability to create, configure, and compare multiple instances useful.
    - I found the ability to copy instances useful.
    - I found the ability to reset instances to initial configuration useful.
    - I have used the output graphs frequently during my use of the system.
    - I could easily extract the information relevant to me from the graphs.
    - I regularly read the exact numerical values of a plot using the mouse-over function.
    - I found the visual highlighting of the simulated components of the alveolus when the cursor was over a parameter group helpful.
    - I found the visual highlighting distracting.
    - I have disabled visual highlighting for most of the time I used it.
    - I found the ability to reset the simulation time helpful.
    - The mouse-over tooltips help assisted me in using the system.
2. On which device or which version(s) of the system did you use? You can use detailed information and multiple selections if, for example, you used multiple usage paths. Detailed information about the operating system (for example, ”Windows 10 version 1903”, ”macOS 10.13”), as well as the device (for example, processor (i5-5700) or graphics card (GeForce 2 MX), or computer model (MacBookPro Late 2015)) or browser (for example, Firefox 83.0, Safari 12) is helpful, especially if problems occurred.
    - Windows Desktop (.exe)

- macOS Desktop (.app)
- Linux Desktop
- Browser (WebGL)
- iOS Tablet
- Android Tablet
- iOS Smartphone
- Android Smartphone

3. On a scale of 1 (strongly disagree) to 7 (strongly agree), please rate the extent to which you agree with the following statements regarding the system.
   - The system responded to my input immediately.
   - Animations were smooth and without annoying leaps.
   - The performance of the system affected my desired use.
4. Which benefits do you see in this system compared to a traditional text book?
5. For which topics from your previous studies would you have appreciated a comparable application?
6. Assuming you'll be teaching physiology - Could you imagine integrating this application into your own teaching?
7. Could you imagine using a similar system on an appropriate topic in your classes (or a similar event)?
8. Please share general comments, suggestions and feedback.

S2.2 Supplementary evaluation of case study

Participants could be divided into two groups with previous knowledge of level 1 and level 2. The scores of their answers on subject-specific tasks differed only minimally (Figure S1). The evaluation of customized questions on the use of *Alvin* is summarized in Figures S2 to S8.

REFERENCES

Gehr, P., Bachofen, M., and Weibel, E. R. (1978). The normal human lung: ultrastructure and morphometric estimation of diffusion capacity. *Respiration Physiology* 32, 121–140. doi:10.1016/0034-5687(78)90104-4

Hurtienne, J. and Naumann, A. (2010). QUESI - A Questionnaire for Measuring the Subjective Consequences of Intuitive Use. In *R. Porzel, N. Sebanz and M. Spitzer (Eds.), Interdisciplinary College 2010* (Sankt Augustin: Fraunhofer Gesellschaft), chap. Focus Theme: Play, Act and Learn. 536

Mercer, R. R., Russell, M. L., and Crapo, J. D. (1994). Alveolar septal structure in different species. *Journal of Applied Physiology (Bethesda, Md.: 1985)* 77, 1060–1066. doi:10.1152/jappl.1994.77.3.1060

Moshagen, M. and Thielsch, M. T. (2021). Facets of visual aesthetics. *International Journal of Human-Computer Studies* 268, 689–709. doi:10.1016/j.ijhcs.2010.05.006

Mühlfeld, C., Weibel, E. R., Hahn, U., Kummer, W., Nyengaard, J. R., and Ochs, M. (2010). Is Length an Appropriate Estimator to Characterize Pulmonary Alveolar Capillaries? A Critical Evaluation in the Human Lung. *The Anatomical Record* 293, 1270–1275. doi:10.1002/ar.21158

Ochs, M., Nyengaard, J. R., Jung, A., Knudsen, L., Voigt, M., Wahlers, T., et al. (2004). The number of alveoli in the human lung. *American Journal of Respiratory and Critical Care Medicine* 169, 120–124. doi:10.1164/rccm.200308-1107OC

Pierigè, F., Serafini, S., Rossi, L., and Magnani, M. (2008). Cell-based drug delivery. *Advanced Drug Delivery Reviews* 60, 286–295. doi:10.1016/j.addr.2007.08.029

TABLES AND FIGURES

Parameter	Value from literature	Reference	Blender model
Alveolar diameter [µm]	225	(Mercer et al., 1994)	225
Alveolar volume [µm³]	$4.2 \cdot 10^6$	(Ochs et al., 2004)	$5.4 \cdot 10^6$
Alveolar surface area [µm²]	121000	(Mercer et al., 1994)	150000
Capillary volume [µm³]	808000	abstracted from (Gehr et al., 1978; Ochs et al., 2004)	787000
Capillary surface area [µm²]	479000	abstracted from (Gehr et al., 1978; Ochs et al., 2004)	335000
Capillary radius [µm]	3.15	(Mühlfeld et al., 2010)	4.28
Capillary segment length [µm]	5.92	(Mühlfeld et al., 2010)	8.62

Table S1. The visual three-dimensional model of an alveolus was created in Blender®. The size ratios were based on morphometric values from the literature.

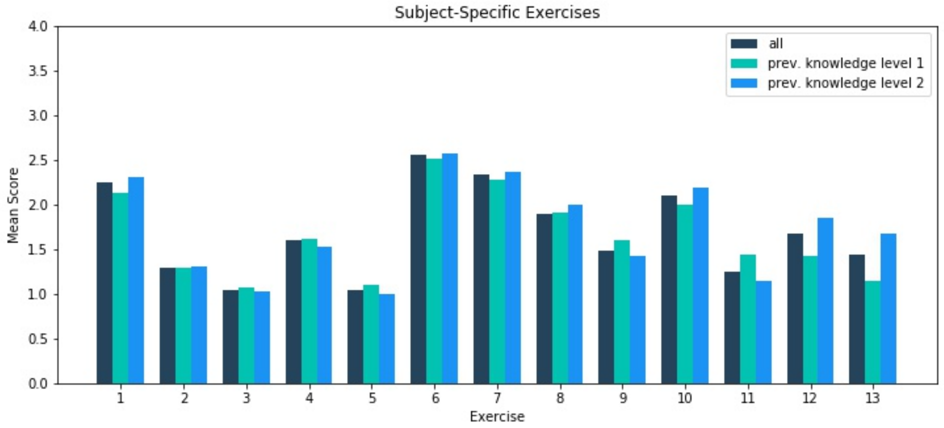

Figure S1. Participants were asked to solve 13 subject-specific exercises with the help of *Alvin*. Responses were scored 1 - correct, 2 - partially correct (e.g. subsequent faults), 3 - unclear to 4 - incorrect. Of the N = 73 participants, N = 31 were assigned to the group with previous knowledge level 1 (attendance of physiology lecture and / or knowledge from school or training). N = 34 participants were assigned to the group with previous knowledge level 2 (attendance of physiology lecture and additional literature).

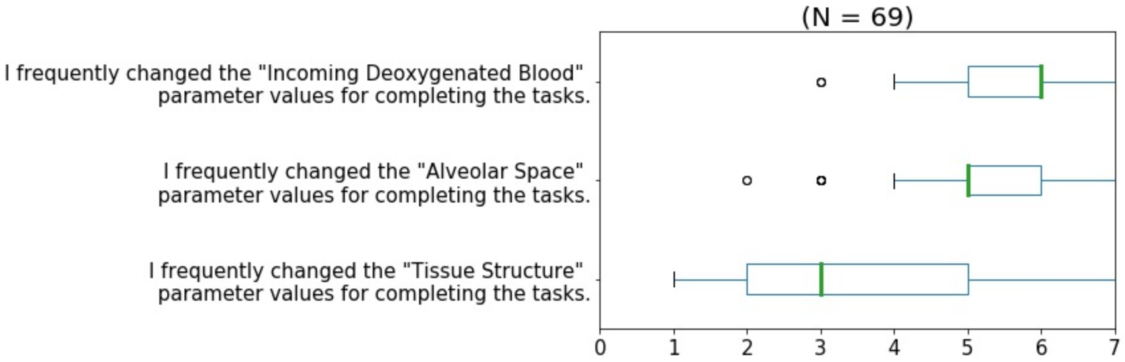

Figure S2. Evaluation of custom questions on the use of the parameter menu in *Alvin*.

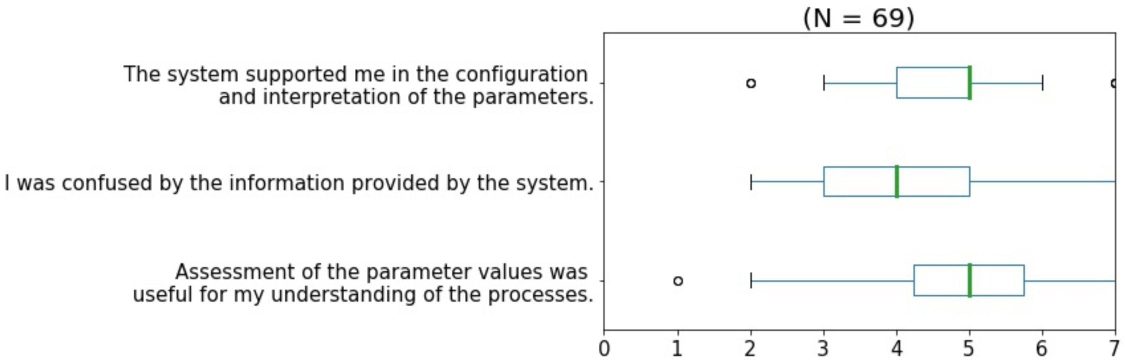

Figure S3. Evaluation of custom questions on the use of additional information on parameters in *Alvin*.

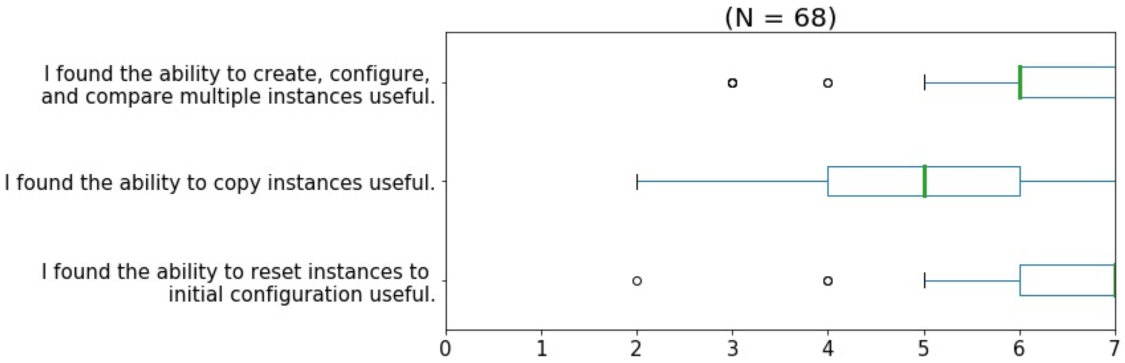

Figure S4. Evaluation of custom questions on the use of simulation instances in *Alvin*.

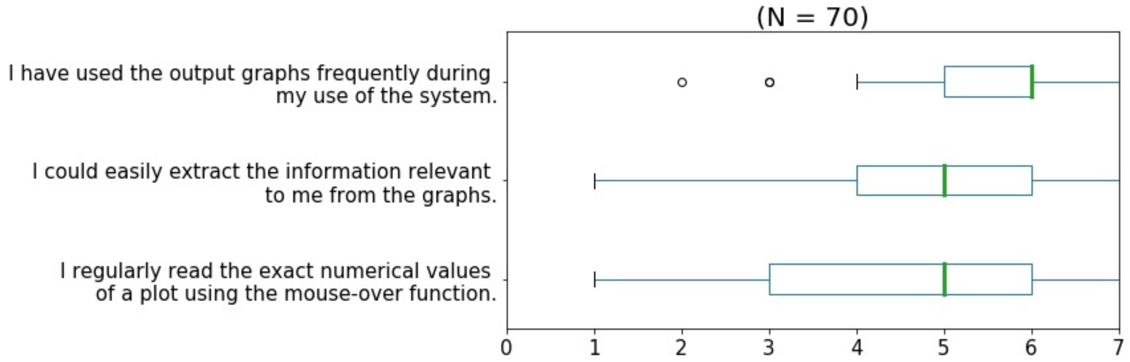

Figure S5. Evaluation of custom questions on the use of the dynamic graphs in *Alvin*.

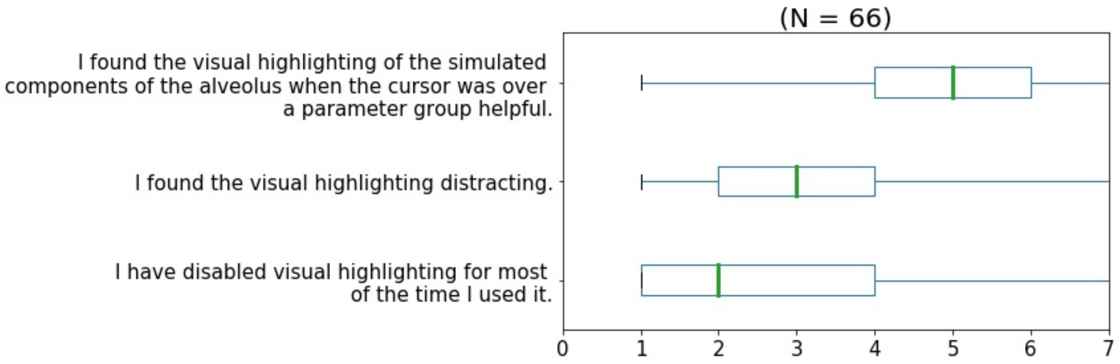

Figure S6. Evaluation of custom questions on the visual highlighting in *Alvin*.

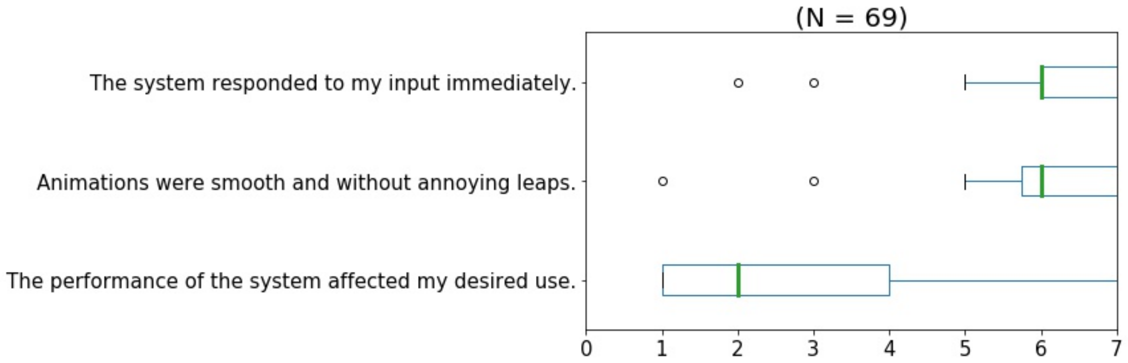

Figure S7. Evaluation of custom questions on the performance of *Alvin*.

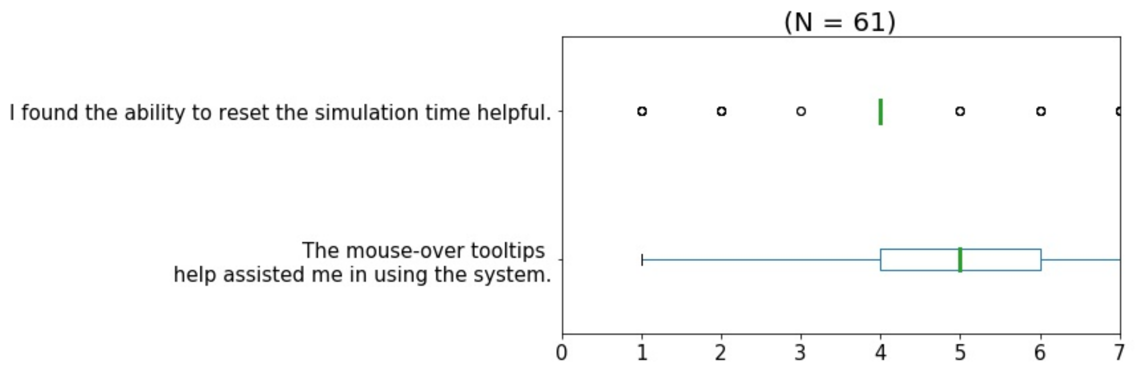

Figure S8. Evaluation of other custom questions on the use of *Alvin*.
